# *Helicobacter pylori* Strains and Gastric MALT Lymphoma

**DOI:** 10.3390/toxins9040132

**Published:** 2017-04-08

**Authors:** Pauline Floch, Francis Mégraud, Philippe Lehours

**Affiliations:** INSERM, Univ. Bordeaux, UMR1053 Bordeaux Research In Translational Oncology, BaRITOn, F-33000 Bordeaux, France; pauline.floch@u-bordeaux.fr (P.F.); francis.megraud@chu-bordeaux.fr (F.M.)

**Keywords:** *Helicobacter pylori*, pathogenesis, virulence marker, *cag* pathogenicity island, MALT lymphoma

## Abstract

This article summarizes the main findings concerning *Helicobacter pylori* associated with gastric MALT lymphoma (GML). Considered together, GML strains based on their virulence factor profile appear to be less virulent than those associated with peptic ulcers or gastric adenocarcinoma. A particular Lewis antigen profile has been identified in GML strains and could represent an alternative adaptive mechanism to escape the host immune response thereby allowing continuous antigenic stimulation of infiltrating lymphocytes.

## 1. Introduction

Gastric MALT lymphoma (GML) is the most common marginal zone lymphoma of the digestive tract. The involvement of *Helicobacter pylori* in this lymphoma is now well established and is based on epidemiological, pathological, clinical, and bacteriological evidence [1]. *H. pylori* eradication therapy is now considered the first therapeutic approach for low grade GML [2,3]. Further studies have indeed revealed a regression of GML lesions after antibiotic eradication of the bacteria [4,5]. *H. pylori* eradication allows lymphoma regression in 60% to 90% of patients [6]. If a reinfection occurs, GML reappears and evolves more rapidly because neoplastic cells are already sensitized to *H. pylori* antigens [7].

*H. pylori* infection was the first bacterial infection to be considered as a type I carcinogen (maximum level) for its implication in gastric adenocarcinoma. Since its discovery, extensive research has been devoted to the identification of virulence factors or genetic markers, but *H. pylori* strains associated with GML have been little studied.

We will endeavor in this review to answer one main question: are there *H. pylori* strains which are more capable of inducing GML than others?

## 2. GML and Cytotoxin-Associated Gene A (CagA)

*H. pylori* is perfectly suited to the human stomach with an armamentarium allowing it to withstand stomach acid, move in the gastric mucosa and evade the immune response of the host [8]. The main virulence factors studied in *H. pylori* are those involved in inflammation and cell damage, in particular those encoded in the *cag* pathogenicity island (*cag*PAI) as well as other pro-inflammatory proteins [9]. The *cag*A gene, encoded by the *cag*PAI, is undoubtedly the most studied virulence factor. Inside the host cells, CagA can be phosphorylated and exert cellular effects dependent on tyrosine phosphorylation, but it can also exert cellular effects independent of CagA phosphorylation, namely IL-8 secretion [10,11,12]. CagA positive strains are considered to be more virulent and are associated with peptic ulcers and gastric adenocarcinoma [13,14] while their association with GML is contradictory.

The results of various studies based essentially on serological data (detection of CagA antibodies) have not been consistent. Some have shown an association between CagA positive strains and the occurrence of GML [15,16,17,18,19,20,21,22] and, more importantly, the prevalence of CagA positive strains in diffuse large B-cell lymphoma (DLBCL) [17,21] (Table 1).

Indeed, CagA is able to translocate into human B lymphocytes in vitro via the type 4 secretion system encoded by the *cag*PAI [23,24]. Once in the cytoplasm, the protein binds to SHP-2, which stimulates B lymphocyte proliferation and inhibits apoptosis via the regulation of intracellular pathways, including the activation of endoplasmic reticulum kinases 1 and 2 (ERK 1 and ERK 2) and p38 MAP kinase (MAPK) and an increase in the expression of Bcl-2 and Bcl-XL [24,25]. The correlation between CagA expression and the expression of SHP-2, ERK, MAPK, Bcl-2 and Bcl-XLT has been confirmed in humans [26]. In transgenic mice ubiquitously expressing CagA, leukocytosis is induced as well as myeloid leukemias and B lymphomas via the deregulation of SHP-2, which is in favor for its role in the pathophysiology of GML. This activity would be dependent on CagA phosphorylation [27]. CagA also acts by inhibiting the accumulation of p53, an important regulator of apoptosis and tumor suppressor, and thus allows B-cells to evade apoptosis leading to the accumulation of genetic mutations [23]. In contrast to CagA activity in the deregulation of SHP-2, this inhibitory effect of CagA would be independent of its phosphorylation [23]. CagA also has an inhibitory activity on B lymphocyte proliferation via the suppression of the JAK-STAT signaling pathway which would allow the bacterium to avoid a specific immune response [23].

Concerning the expression level, Kuo et al., [28] detected the CagA protein in malignant B cells in half of the GML patients studied. They observed that patients developing GML and infected by a CagA positive strain responded significantly faster to eradication than those infected by a CagA negative strain [28].

It is now established that *H. pylori* strains expressing the CagA protein are not associated with low grade GML, but rather with gastric DLBCL [17,21,29,30]. Serological and strain analyses showed that the prevalence of CagA was significantly higher in gastric DLBCL (approximately 75%) than in GML (37.8% to 44.8%) [17,21]. CagA and CagA signaling molecule expression in tumor cells could be markers of *H. pylori* dependence on gastric DLBCL [30]. The results of the study conducted by Lehours et al., based on a large collection of well-characterized MALT strains, are consistent with the absence of CagA association with GML. The absence of CagA in about half of the GML strains studied indeed suggests the existence of other mechanisms in lymphomagenesis. These strains are probably less pro-inflammatory than strains associated with peptic ulcer or gastric adenocarcinoma. This was confirmed in vitro during co-culture experiments with a gastric epithelial cell line, namely AGS. GML strains and, in particular, the *cag*PAI negative ones did not have a particular pro-inflammatory potential in this experimental system and would probably produce no other major pro-inflammatory factor than those encoded by the *cag*PAI [10].

## 3. GML and Vacuolating Cytotoxin A (VacA)

The vacuolating cytotoxin VacA was named for its ability to induce the formation of vacuoles in some cell lines in vitro. The protein of 140 kDa is encoded by the *vac*A gene. All *H. pylori* strains have a copy of this gene, but only 50% possess the vacuolating ability in vitro. This is explained by its polymorphism, the variable level of gene transcription [31] and the level of the protein’s secretion [32]. Three major regions of diversity (s, i and m) in the *vac*A sequence gene have been described: the signal s sequence is characterized by four different families (s1a s1b, s1c, s2), the m central region by three families (m1, m2a, m2b) and the intermediate region i by three families (i1, i2, i3). Each gene has a combination of these different sequences which leads to numerous alleles and determines the activity of the toxin [33].

Epidemiological studies have shown a correlation between these alleles and the risk of developing a gastroduodenal disease. The risk of gastric adenocarcinoma or peptic ulcer development is increased in people infected with strains carrying s1, m1 or i1 alleles compared to those infected with s2, m2 or i2 strains [33,34,35].

It has also been shown that VacA induces epithelial cell apoptosis both in vitro and in vivo [36,37]. VacA penetrates inside the mitochondria, leading to a release of cytochrome C and thereby activating pro-apoptotic signaling pathways [38].

The combination of *vac*A alleles with GML was also studied. Indeed, the *vac*Am2 allele, corresponding to the less biologically active strains (the less vacuolating in vitro, the less pro-apoptotic and less biologically active in vivo), predominates in GML strains [39,40] (Table 1). The *vac*A s1m1 genotype (corresponding to a high level of cytotoxin production) was correlated to the presence of *cag*A and *cag*E, suggesting that these virulence genes are closely associated (as already described in *H. pylori* strains leading to the other diseases), however, the evolution toward GML remain to be elucidated.

Finally, VacA was shown to exhibit in vivo anti-lymphoproliferative properties, especially on T cells. This should be interpreted with care, in line with GML pathogenesis. VacA inhibits the activation and proliferation of B and T lymphocytes [41,42,43] and could therefore interfere with the antigen presentation of B-cells [44].

## 4. Other Virulence Factors

The bacterial adherence capacity is essential for good colonization and persistence of the infection. *H. pylori* multiplies in the gastric mucus and the surface of epithelial cells is reached by a small proportion of bacteria. The expression of adhesins allowed them to adhere [45]. *H. pylori* must be able to adhere to gastric epithelial cells to avoid being eliminated by the gastric peristalsis and mucus renewal [46]. Several adhesins have been described, with the most studied being BabA (Blood group antigen binding adhesin) and SabA (Sialic acid-binding adhesin). These proteins bind to Lewis antigens, which are similar to those of blood groups and are present on the surface of gastric epithelial cells [47]. There are two alleles for the *bab*A gene; *bab*A1 and *bab*A2. *H. pylori* strain sequences could contain one, two or multiple copies of the *bab*A gene [48]. The *bab*A2 strains are associated with ulcers and adenocarcinoma [49,50]. Recognition of SabA by neutrophils allows their activation, and thus a release of radical oxygen and nitrogen species, inducing epithelial lesions [51]. The major adhesins, BabA and SabA, and the different outer membrane proteins modulate their expression depending on the environmental context [52]. Thus, the BabA protein hence could be modulated by phase variation and antigenic variation in vivo, to facilitate adherence to the epithelium and to permit chronic infection [53].

Lehours et al. studied the presence of *H. pylori* virulence factors (*cag*A, *cag*E, *vac*A alleles, *hop*Q, *ice*A and *bab*A) and functional status of both *sab*A and *hop*Z genes, in 43 GML strains compared with 39 strains isolated from gastritis [40]. None of these genes were associated with GML when considered individually. However, the gene group comprised of *ice*A1 allele, *sab*A and *hop*Z were identified in strains with a ten times higher risk of developing GML than strains associated with gastritis. The low prevalence of these strains among GML strains, however, made it a low sensitivity marker (Table 1).

## 5. GML and Lipopolysaccharide (LPS) Antigens

The O chain of *H. pylori* LPS has a similar composition to the Lewis X type antigens (Le^x^) or the Lewis Y (Le^y^) blood group, also found in gastric epithelial cells [54]. This bacterial mimicry results in an escape from the immune response; *H. pylori* is no longer recognized as a non-self which promotes colonization and contributes to chronic infection [55,56]. Moreover, this mimicry is involved in a phenomenon of autoimmunity leading to gastric atrophy [57]. The nature of the Lewis antigen expressed by the *H. pylori* LPS determines the interaction with dendritic cells via a C-type lectin called DC-SIGN, present on the surface of dendritic cells [58], that could influence the pro-inflammatory response. Lewis negative strains escape the association with DCs and induce a Th1 response, while strains expressing Le^x^ and or Le^y^ bind to DC-SIGN, resulting in the production of a IL-10-Treg associated response and the obstruction of a Th1 response.

LPS antigens expressed by GML strains were studied by our group [59]. *cag*PAI negative GML strains strongly expressed Le^y^ antigens. These Le^y^ antigens were associated in the past with autoimmune manifestations, suggesting a component of this type in the pathogenesis of GML. The association between Lewis antigen expression and disease status is not modified by *vac*A genotypes. In conclusion, a particular Lewis antigen profile has been identified in *cag*PAI negative MALT strains, which could represent an adaptive mechanism to the host response and participate in MALT lymphomagenesis (Table 1).

The chronicity of *H. pylori* infection is believed to be essential in the context of gastric MALT lymphoma. LPS is an important effector of the TLR4 among various Gram-negative bacteria. However, *H. pylori* LPS evades TLR4 recognition, which therefore plays an important part in this “camouflage” strategy [8]. According to Suarez et al., an antigenic source of autoimmunity is provided by the chronic microbial antigenic stimulation observed during persisting *H. pylori* infection. This phenomenon leads to sustained B-cell stimulation, thus favoring lymphoid transformation and lymphoma development [60].

## 6. GML Strains and Genomic Data

Subtractive hybridization, a technique based on multiple steps of DNA-DNA hybridization, PCR, cloning and sequencing, was used to identify specific genetic markers of GML strains. This technique allows the identification of genes or sequences present in a strain of interest (called the tester strain) in comparison to a control strain (called the driver). It was originally used to identify the *cag*PAI [61].

One marker, ORF JHP950 (according to the strain J99 annotation), was identified in GML strains. It belongs to the so-called *H. pylori* plasticity zone [62,63]. This area was not initially considered as a pathogenicity island *sensus stricto*, but rather as a large genomic island. However, JHP950 is located close to ORF JHP947, which has been associated with strains isolated from patients with gastric adenocarcinoma [63]. A significant association of JHP950 with *ice*A1 and *sab*A virulence genes was also found in GML isolates [64] (Table 1). JHP950 ORF encodes a protein with no specific function, which therefore poses a problem to integrate its role in the pathogenesis of GML. Only complementary approaches such as reverse genetics or even proteomics would help to answer this question. Nevertheless, ORF JHP950 is the first and only genetic marker to date that may be used to screen high-risk GML strains.

In a study performed by Thiberge et al., 43 DNAs extracted from GML strains were hybridized to high-density membranes containing a selection of 248 non-ubiquitous genes (the flexible part of the *H. pylori* genome known at that time) and 50 ubiquitous genes (the stable part) [65] (Table 1). A homogeneous subpopulation of strains exclusively composed of *cag*PAI negative GML strains was identified by hierarchical cluster analyses of the DNA hybridization values. These *cag*PAI negative strains therefore appeared more closely together than others, suggesting again that the GML strains share common genetic background.

This study motivated the same group to sequence and fully annotate the genome of one of these *cag*PAI negative strains. This strain, named B38, represented the smallest published genome (1,576,758 base pairs containing 1528 CDSs) compared to the six previously released *H. pylori* genomes at that time (i.e. J99, 26695, HPAG1, P12, G27 and Shi470) [65]. It contains the *vac*A s2m2 allele and lacks the genes encoding the major virulence factors (absence of *cag*PAI, *bab*B, *bab*C, *sab*B, and *hom*B). A small prophage was identified in this strain. The presence of prophages was further confirmed in approximately 20% of *H. pylori* strains; there was with no association with GML, but with phylogeographic groups of *H. pylori* [66,67].

More recently, three *H. pylori* strains isolated from patients with GML were sequenced by Wang et al. [68]. Nine genes shared by these three strains and absent in five *H. pylori* strains isolated from gastritis and ulcer were identified by whole-genome comparison. Many gene substitutions, deletions and insertions were also revealed in these three strains. Further investigations are needed to understand the implication of these genetic variabilities in gastric lymphomagenesis (Table 1). Knowledge of the genome sequences of GML strains could open new perspectives to explore the contribution of virulence determinants in the physiopathology of *H. pylori* infection.

## 7. Conclusions

No specific virulence factor has been identified yet in GML-associated strains to explain gastric lymphomagenesis. The situation is very different from gastric adenocarcinoma’s associated strains, where the molecular effects induced by the *cag*PAI are now well characterized and linked to gastric carcinogenesis. Compared to strains associated with peptic ulcer or gastric adenocarcinoma, GML strains based on their virulence factor profile appear to be less pro-inflammatory. They can indeed be considered amongst the lowest producers of VacA cytotoxin, which could be a strategy to modulate T cell functions in vivo. Based on their genetic content and LPS profile, *cag*PAI negative GML strains seem to be closely related, even if no major new virulence factor has been identified in this group of strains. Some genetics variabilities in GML-associated strains have been identified, but further investigations are needed to understand their potential implication in GML development. In vivo models of GML are under development and could bring new data on the nature of stimulating and recognized antigens involved in GML pathogenesis in the near future. Are there *H. pylori* strains that are more capable of inducing GML than others? The answer is probably “no.” The information gained over the past 15 years on GML-associated strains suggests that the key point in gastric lymphomagenesis should be investigated elsewhere, probably in predisposing host factors.

## Figures and Tables

**Table 1:** Specificities of gastric MALT lymphoma strains.

Virulence Factors	Association with Gastric MALT lymphoma	References
CagA	Controversial implication. *cag*PAI present in only 50% of GML isolated strains. Association with high grade lymphoma questionable.	[17,21,28,40]
VacA	*vac*Am2 allele predominant in GML strains (genotype associated with the lowest biological activity)	[39,40]
BabA	No association with GML.	[40]
SabA		
Other adhesins		
Lipopolysaccharide (LPS) antigens	*cag*PAI negative GML strains expressed Le^y^ antigens, previously associated with autoimmune manifestations. Strategy to escape to host response?	[55,59]
Genetic markers		
Gene group comprised of *ice*A1 allele, *sab*A and *hop*Z	Association with the risk of GML development (low sensitivity marker)	[40]
ORF JHP950	ORF encoding a protein with no specific function, association with *ice*A1 and *sab*A virulence genes. The first and only genetic marker of GML strains.	[64]
DNA array	GML strains share common genetic background.	[65]
Genome sequencing	Further studies are needed to understand biological significance of genetic variabilities	[65,66,68]
